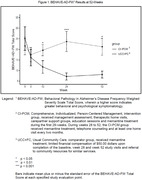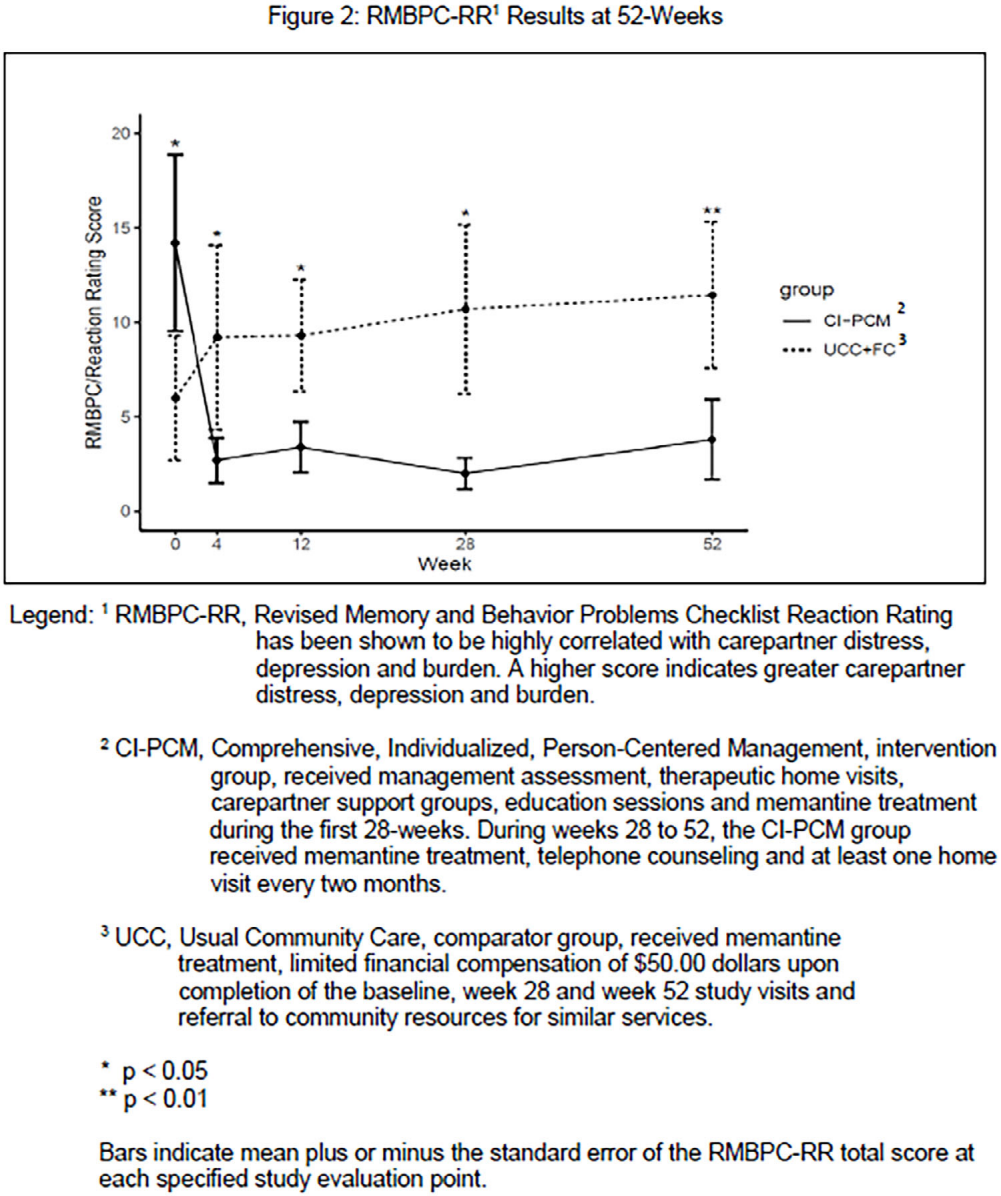# Nonpharmacologic Management of Behavioral and Psychological Symptoms of Dementia in Community‐Residing Persons with Moderately‐Severe Alzheimer’s Disease: One Year Results from a Randomized Controlled Trial

**DOI:** 10.1002/alz.090018

**Published:** 2025-01-09

**Authors:** Sunnie Kenowsky, Yongzhao Shao, Sloane Heller, James B. Golomb, Alok Vedvyas, Gianna Dafflisio, Qiao Zhang, Harris Torossian, Martin J. Sadowski, Thomas Wisniewski, Arjun V. Masurkar, Barry Reisberg

**Affiliations:** ^1^ Center for Cognitive Neurology, New York University Langone Health, New York, NY USA; ^2^ Alzheimer’s Disease Research Center, New York University Langone Health, New York, NY USA; ^3^ New York University Grossman School of Medicine, New York, NY USA; ^4^ NYU Langone Health, New York, NY, USA,, New York, NY USA; ^5^ Division of Critical Care and Hospital Neurology, Columbia University Irving Medical Center, New York, NY USA; ^6^ Penn State College of Medicine, Penn State Health Milton S. Hershey Medical Center, Hershey, PA USA; ^7^ New York University Langone Health, New York, NY USA; ^8^ McGill University Centre for Studies in Aging, Montreal, QC Canada

## Abstract

**Background:**

Behavioral and psychological symptoms of dementia (BPSD) occur frequently in persons with Alzheimer’s disease (PAD). They cause suffering, institutionalization, carepartner distress, depression, burden, and decreased PAD‐carepartner quality of life.

Brexpiprazole approval advanced the AD treatment armamentarium. However, pharmacologic treatment options have limited effectiveness and may have serious adverse effects. Therefore, nonpharmacologic interventions are recommended as first‐line treatments. Nevertheless, much still needs to be accomplished to ameliorate PAD‐carepartner outcomes including improving access to implementable, comprehensive, individualized, effective care and trained professionals.

We conducted a 28‐week, randomized, controlled, clinician‐blind, parallel group study (Reisberg…Kenowsky, *Dement Geriatr Cogn Disord*. 2017), in which moderately‐severe PAD‐carepartner dyads (N = 20) received memantine and a comprehensive, individualized, person‐centered management program (CI‐PCM, n = 10) or usual community care (UCC, n = 10), followed by a 24‐week extension study.

In the 28‐week study, carepartners learned to identify BPSD and their causes, communicate and engage PAD effectively, manage and treat medical issues and pain, meet PAD needs, employ behavioral and psychological strategies to manage and prevent BPSD, and techniques to restore PAD abilities and skills. We report 52‐week behavioral outcomes.

**Methods:**

All 28‐week study subjects entered the 24‐week extension during which one UCC subject died. CI‐PCM carepartners implemented training and tools learned in the 28‐week study. Minimal assistance by the AD care specialist consisted of every two‐month home visits and ad hoc telephone consultations. BPSD were assessed using the Behavioral Pathology in Alzheimer’s Disease Frequency Weighted severity scale (BEHAVE‐AD‐FW). The Revised Memory and Behavior Problems Checklist, Reaction Rating (RMBPC‐RR) was used to assess carepartner reaction to BPSD. P values for difference from baseline to 52‐weeks were determined using the Wilcoxon‐Mann‐Whitney test. Cohen’s d was calculated to estimate effect size.

**Results:**

BEHAVE‐AD‐FW showed significant improvement in CI‐PCM subjects for severity and frequency of total BPSD (Figure 1, p = 0.01, Cohen’s d = 1.203), paranoid and delusional ideation (p = 0.011) and agitation (specifically, aggressivity, p = 0.041). CI‐PCM carepartner distress, depression and burden significantly decreased (RMBPC‐RR, Figure 2, p = 0.004, Cohen’s d = 1.45).

**Conclusions:**

CI‐PCM is an effective, readily implemented program that significantly decreased total BPSD, aggressivity, and paranoid and delusional ideation in moderately‐severe PAD and significantly improved carepartner distress, depression and burden.